# Errata

**Published:** 2005-01

Because the study “Threshold of Trichloroethylene Contamination in Maternal Drinking Waters Affecting Fetal Heart Development in the Rat” (Johnson et al. 2003) was a long-term and continuous study, the authors compiled the data from controls of several treatment groups. The control “sets” were statistically analyzed comparing the data to each other before being combined. The authors opine that the control values were statistically consistent across and throughout all the treatment groups. Using the control data in a cumulative manner increased the generalizability of the data, which purports to demonstrate the background rate and variability around rate estimates. The larger sample size somewhat increased statistical power without the inappropriate use of further valuable animal resources.

Table 1 presents the date ranges of experimental treatment and the coinciding control treatments. Each treatment exposure had a corresponding control group. Also, because of the more detailed information on competing financial interests now included in EHP’s Instructions to Authors, the authors now report that S.J. Goldberg served as an expert witness for a plaintiff in a judicial hearing in 1997. As previously stated in a prior letter to the editor (Johnson et al. 2004), at all times throughout this research, the authors were free to design, conduct, interpret, and publish the research without compromise by any controlling sponsor as a condition of review or publication.

In “Environmental Health Disparities: A Framework Integrating Psychosocial and Environmental Concepts” by Gee et al. [ Environ Health Perspect 112:1645–1653 (2004)], the title of Figure 1 should be “Stress–exposure disease framework for environmental health disparities.”

## Figures and Tables

**Table 1:** Control versus TCE treatment groups and dates of exposure.

Control		TCE		
Fetuses/mothers*^a^*	Dates	Dose	Fetuses/mothers	Dates
135/15	14 Jun 1989–10 Oct 1992	1,100 ppm	105/9	29 Jun 1989–12 Mar 1990
155/13	11 Dec 1992–20 Oct 1993*^a^*	1.5 ppm	181/13	29 Dec 1989–26 Dec 1990
62/6	15 Apr 1994–23 May 1994*^a^*			
120/10	6 Jul 1994–7 Jul 1995	2.5 ppb	144/12	6 Jun 1995–13 Jun 1995
134/11	18 Jul 1995–6 Oct 1995	250 ppb	110/9	5 Jul 1995–21 Jul 1995

^a^The total number of control rat fetuses/mothers was 606/55.

^b^Other studies that coincided with these control groups were carried out during December 1989–June 1995 [e.g., metabolites that were reported in other articles (Johnson et al. 1998a, 1998b).

## References

[b1-ehp0113-a0018b] Johnson PD, Dawson BV, Goldberg SJ (1998a). Cardiac teratogenicity of trichloroethylene metabolites. J Am Coll Cardiol.

[b2-ehp0113-a0018b] Johnson PD, Dawson BV, Goldberg SJ (1998b). A review: trichloroethylene metabolites: potential cardiac teratogens. Environ Health Perspect.

[b3-ehp0113-a0018b] Johnson PD, Dawson BV, Goldberg SJ, Mays MZ (2004). Trichloroethylene: Johnson et al.’s Response [Letter]. Environ Health Perspect.

[b4-ehp0113-a0018b] Johnson PD, Goldberg SJ, Mays MZ, Dawson BV (2003). Threshold of trichloroethylene contamination in maternal drinking waters affecting fetal heart development in the rat. Environ Health Perspect.

